# Point-of-Care Arterio-Venous Fistula Ultrasound in the Outpatient Hemodialysis Unit—A Survey on the Nurses’ Perspective

**DOI:** 10.3390/nursrep14010027

**Published:** 2024-02-04

**Authors:** Iulia Grosu, Oana Stirbu, Adalbert Schiller, Florica Gadalean, Flaviu Bob

**Affiliations:** 1 Centre for Molecular Research in Nephrology and Vascular Disease, Faculty of Medicine, Victor Babeș University of Medicine and Pharmacy, 300041 Timișoara, Romaniagadalean.florica@umft.ro (F.G.);; 2 Division of Nephrology, Department of Internal Medicine II, County Emergency Hospital Timisoara, Victor Babeș University of Medicine and Pharmacy, 300041 Timișoara, Romania; 3 B Braun Avitum Dialysis Centers, 300417 Timișoara, Romania; 4 Department of Nephrology and Dialysis, Arad County Hospital, 310158 Arad, Romania

**Keywords:** arterio-venous fistula, ultrasound, hemodialysis, HD nurse, point-of-care ultrasound

## Abstract

The preservation of complication-free arterio-venous fistulas (AVF) for long-term hemodialysis (HD) use is associated with better overall patient outcomes, which is why this is a current goal in any HD center. Point-of-care ultrasound (POCUS) for in-center AVF assessment has proven its benefits in the identification of vascular access (VA) complications and as an additional tool to avoid blind cannulation. The current study aims to assess the change in the HD nurses’ perceptions regarding AVF POCUS use in the HD center. The nursing staff anonymously answered a Likert scale questionnaire with five questions related to various aspects of AVF POCUS utility shortly after the technique had been implemented and at a 5-year follow-up. The results showed an overall positive attitude toward this method, both at implementation and at follow-up, with no statistically significant score changes for four out of the five items assessed. However, we found a statistically significant reduction in the nurses’ cannulation confidence scores at the 5-year follow-up (*p* < 0.01). Overall, AVF POCUS implementation is regarded as a useful tool, with major benefits both for the patient and for the medical team. The current study results aim to support the introduction of AVF POCUS assessment as a standard practice from the nursing staff’s viewpoint. This study was not registered.

## 1. Introduction

Arterio-venous fistulas (AVF) are regarded as the “gold standard” for vascular access in chronic hemodialysis (HD) patients [1] due to their extended use and association with overall lower morbidity and mortality [2]. Moreover, AVFs have lower complication rates than other vascular access (VA) types, such as central venous catheters (CVCs) or arteriovenous grafts (AVGs) [3], and they are associated with a better quality of life [4]. 

Even though maintaining long-term AVF patency is a target in the HD setting, vascular access complications occur frequently. AVF complications that are commonly encountered in clinical practice include the following: stenosis, thrombosis, aneurysms, steal syndrome, and insufficient maturation. The current recommendations regarding scheduled ultrasound (US) monitoring of asymptomatic AVFs do not favor regular ultrasound scans [1]. However, if an AVF complication occurs, detected either upon physical examination or due to impaired HD adequacy, the Society of Vascular Surgery Guidelines 2018 recommend performing a Duplex US assessment as soon as possible as a first-line investigation [4].

In the clinical setting, HD nursing staff face difficulties when needling a new, poorly matured AVF, which may result in miscannulations and hematomas. These events lead on many occasions to a HD session’s inadequacy, abortion, and even impaired VA functionality. Furthermore, the cannulation skills of the HD nurse are also important factors to consider. This is a particularly important aspect because the HD population is becoming increasingly older, with the current literature supporting the fact that patients over 65 years old have an increased risk of maturation failure [5]. Other situations in which additional help from a US technique may be considered are as follows: identification of cannulation sites to avoid area puncture; identification of needling sites in difficult, tortuous AVFs; and identification of cannulation complications (hematomas, thrombosis, and stenosis), resulting in an immediate course of action [6].

Point-of-care ultrasound (POCUS) of AVFs requires either a regular US machine moved to the patient’s bedside or a handheld US device with a wide linear 5–13 MHz transducer, a sterile US gel, and a transducer sheath if cannulation or US-guided procedures are involved. US-guided cannulations may be performed by a single operator (holding both the probe and the needle) or by two operators (one holding the probe and the other inserting the needle). The probe may be placed either longitudinally, so that the trajectory of the entire needle can be observed as it advances, or it may be placed transversally, in which case only the tip of the needle will be seen on the US screen [7]. The goal of US-guided cannulations is mainly to prevent AVF infiltrations but also to ensure that the vessel is successfully punctured. Mild AVF infiltrations may occur in 50% of all AVFs, leading to a higher need for further tests and interventions and even leading to AVF thrombosis [8]. Niyyar et al. [9] designed a pilot protocol of US guidance for the cannulation of all new AVFs and achieved a 1.7% AVF infiltration rate, thus showing the effectiveness of the method for preventing these complications.

Even though technology has evolved and there is a wide range of devices that may be used, only the Canadian Association of Nephrology Nurses and Technologists’ (CANNT) group provides a clear recommendation of including POCUS as a method of improving cannulations alongside physical examination [10]. The caveat with POCUS is that it requires either physician or nurse training, which is currently not an international standard practice.

A study by Schoch et al. [11] raised the issue that a lack of confidence in the method, a lack of experience, and a lack of time were the main contributing factors for the reluctance to use POCUS more in the clinical setting. Since the nursing staff’s approach regarding AVF cannulation and potential complications is crucial in the HD unit, our present study aims to assess the impact of AVF POCUS implementation on nurses’ viewpoints. Therefore, potential changes in nursing practice may highlight and reiterate the need for AVF POCUS training and application as a standard practice.

## 2. Materials and Methods

### 2.1. Study Setting

The current study was designed as a single-center survey, repeated after a period of 5 years. It involved two sets of questionnaires assessing the nurses’ perceptions of vascular access management quality improvement shortly after POCUS implementation and after 5 years, when it had already become standard practice. The eligibility criteria for initial and follow-up study inclusion were as follows: (1) working full-time in the dialysis unit; (2) being a registered nurse who performs cannulations in the dialysis unit.

The attending nephrologists had received training to perform AVF-US in January 2018. The nursing staff were acquainted with the POCUS method via a theoretical course organized shortly afterwards, which also included information regarding AVF complications, cannulation techniques, and good standard practices, as recommended by the acting guidelines. The POCUS program was implemented in February 2018 in the HD unit using a portable Edan Acclarix 4 × 4 ultrasound machine and a L12-5HQ linear transducer. The US assessments were performed pre/post-dialysis but also during the dialysis session (upon request) by the attending nephrologist, who was in each case assisted by the nurse. The US-guided cannulation procedure was performed by the nurse with the nephrologist’s aid. The skin was disinfected, the probe was dressed in a sterile sheath, and sterile conductor gel was used to not risk contaminating the cannulation site. The transducer was held longitudinally by the nephrologist, indicating the exact needle insertion site to the nurse. The nurse inserted the needle, having a direct visualization of its progression in the vessel on the US machine screen. Once the needle tip reached the mid-section of the vessel, it was secured in place.

### 2.2. Survey Design

In July 2018, the nursing staff were invited to respond to an anonymous 5-item Likert survey, with a response scale ranging from 1 to 5 as follows: 1—complete disagreement; 2—disagreement; 3—neutral; 4—in agreement; 5—total agreement. The time to reply was 7 days from the initial handout. The survey included a question regarding HD nursing experience (years).

All of the 35 employed nurses participated and responded to the following items (July 2018):

1. AVF POCUS is helpful in the successful cannulation of the new/difficult AVFs.
2. AVF POCUS helps to improve my cannulation technique.
3. AVF POCUS has a positive effect in increasing my confidence in my vascular access cannulation skills.
4. AVF POCUS is a tool for better vascular access teamwork.
5. AVF POCUS is an indispensable tool for AVF care.

After continuing POCUS as standard practice in the HD unit for 5 years when AVF complications/miscannulations/new AVFs occurred, a follow-up survey was issued in July 2023. The questions were the same, with the same Likert response scale range as previously stated, to provide a comparative assessment of the change in the nurses’ perception of AVF POCUS over time. Thirty-four (34) nurses participated in the second survey (July 2023), and the items were the same as in the first survey. The second survey was anonymous as well, and similarly to the first one, it included a question regarding HD nursing experience. Based on the answers provided, 15/34 (44.1%) started their employment after the first survey was issued.

Each nurse gave informed consent to participate in the study. The survey was conducted in accordance with the Declaration of Helsinki and with the approval of the Dialysis Centre (study approval number 33/30.06.2021)

### 2.3. Statistical Analysis

The statistical analysis was performed using the MedCalc version 22.014 program.

The normal distribution of the survey group age and employment duration, as well as the normal distribution of survey responses, was assessed using the Shapiro–Wilk test. Normal distribution was rejected in all questions and accepted for age and employment duration. To assess the differences in the paired items of the questionnaire, we used the Mann–Whitney U test, given the non-normal distribution of the responses. To assess differences between the two groups (initial survey versus follow-up study characteristics), we used independent sample t-tests. A *p*-value of <0.05 was considered statistically significant.

## 3. Results

The first survey included 35 nurses from a single dialysis unit with a mean age of 36.7 ± 11.38 years; males constituted 11.42% (*n* = 4); and the average employment in HD was 6.18 ± 3.78 years. The follow-up study included 34 nurses, aged 38.2 ± 12.45 years, and 8.8% males (*n* = 3), with an average employment in HD of 8.51 ± 6.11 years.

All the nurses were full-time employed in the HD unit (Table 1). All the surveys that were handed out were anonymous. Because there was staff turnover during the 5-year follow-up period, we could appreciate, based on the employment years, that 15/34 (44.1%) nurses had not responded to the initial questionnaire.

### 3.1. Initial Survey Results

At the first statement, “AVF POCUS is helpful in the successful cannulation of the new/difficult AVFs”, 34 nurses (97%) responded with 5, strongly agree, and 1 (3%) of the participants responded with 4, agree, to the question. The second question, “AVF POCUS helps to improve my cannulation technique”, had the following results: 94.3% (*n* = 33) of the nurses answered with 5, strongly agree; 3% (*n* = 1) with 4, agree; and 3% (*n* = 1) with 3, neutral. The third statement was, “AVF POCUS had a positive effect in increasing my confidence in my vascular access cannulation skills”, and 91% (*n* = 32) of the participants responded 5, strongly agree, and 6% (*n* = 2) responded 4, agree (4/5). However, one participant (3%) responded with 1, strongly disagree, to this item (1/5). At the fourth statement, “AVF POCUS is a tool for better vascular access teamwork”, 29 nurses (83%) responded with 5, strongly agree, and 6 participants (17%) responded with 4, agree, that US of AVFs helps the team members to better provide better vascular access care. At the fifth statement, “AVF POCUS is an indispensable tool for AVF care”, 77% (*n* = 27) of the nurses responded with 5, strongly agree, while 20% (*n* = 7) responded with 4, agree, and 1 participant (3%) responded with 3, neutral (*n* = 1).

### 3.2. Follow-Up Survey Results

The 5-year follow-up results for the first statement were as follows: 5, strongly agree. 88% (*n* = 30); 4, agree, 12% (*n* = 4). For the second statement, 82% (*n* = 28) were in full agreement, 12% (*n* = 4) answered with 4, agreement, and 6% (*n* = 2) answered with 3, neutral. For the third statement, 53% (*n* = 18) answered fully agree, 41% (*n* = 14) agreed, while 6% (*n* = 2) were neutral. At the fourth question, 65% (*n* = 22) fully agreed, 24% (*n* = 8) agreed, and 11% (*n* = 4) disagreed. At the final question, 71% (*n* = 24), responded with 5, “fully agree”, and 29% (*n* = 10) answered with 4, “agree”. The comparative side-by-side responses to all the questions (initial and follow-up surveys) are presented in Table 2.

When assessing the differences in the items of the questionnaire using Mann–Whitney U tests, we recorded no statistically significant differences for items 1, 2, 4, and 5 (*p* > 0.05); however, there was a difference, with a lower overall score for the follow-up survey, for question 3 (Table 3).

## 4. Discussions

Maintaining AVF patency and limiting vascular access complications reduces the mortality and hospitalizations of HD patients [12] and improves their quality of life [13,14]. Bearing in mind the importance of the nursing staff in the hemodialysis unit and VA management, the current study aims to bring forward their perceptions on AVF POCUS implementation. Introducing AVF POCUS into dialysis units is not standard practice yet, even though it has been shown in multiple studies to have a beneficial role in successful cannulations and decreasing adverse cannulation events [15]. Similarly, US guidance has been used in other clinical settings regarding vascular cannulations, both venous and arterial. A meta-analysis by Wu et al. [16], including 26 studies, showed that using real-time two-dimensional US when inserting central venous catheters decreased the risk of cannulation accidents such as cannulation failure, arterial puncture, hematoma, or hemothorax. Another relevant meta-analysis by Zawadka et al. [17] regarding subclavian vein cannulation showed better results regarding success and complication-free rates when using the US-guided technique versus the landmark “blind” approach. Similar positive results supporting US-guided radial artery punctures were reported by Gu et al. [18], especially regarding first-attempt success rates. Data regarding the nurses’ perceptions of US guidance for performing different procedures is, however, scarcer. In a study by Blaivas et al. [19] on 321 nurses regarding the perceived potential aid of the US-guided placement of peripheral intravenous lines, the authors described a decrease in needling difficulty after US training sessions.

The statements that were implied in our survey had a gradual approach in order to highlight the multiple aspects in which the use of AVF POCUS may impact the nurses’ activity. These ranged from POCUS as a helpful tool for difficult AVFs to the impact of using the method on cannulation technique improvement, raising confidence levels, and harboring teamwork, and ultimately, whether this method is indispensable in the dialysis unit. We highlight the fact that this is the first study to assess perception outcomes for such a long period of time (5 years), thus excluding the initial excitement a novel method may convey and offering a realistic perspective from the nursing staff.

Regarding the first item, “AVF POCUS is helpful in the successful cannulation of new/difficult AVFs”, we obtained similar, 100% positive (agreement or total agreement) results, both at the initial survey and at follow-up, with no statistically significant differences. This is in accord with studies showing the utility of AVF POCUS-guided cannulations. Studies by Farpour et al. [20] demonstrated the superiority of POCUS-guided cannulation versus blinded cannulation in difficult as well as new fistulas, while the group of Marticorena found a reduced rate of adverse cannulation events when using POCUS in a Canadian group [21].

The second item of the survey, “AVF POCUS helped to improve my cannulation technique”, was also favorably assessed, both initially and upon follow-up, with over 94% answers either in agreement or in full agreement. This is of particular interest from a prospective point of view because correct cannulation techniques lead to fewer AVF complications [22]. One study [23] regarding POCUS needle evaluation during HD sessions suggested that placing the tip of the needle in the middle of the vessel minimized the mechanical trauma of the AVF. On the contrary, if the needle is too close to the anterior or posterior walls due to flow turbulences it will increase the shear wall stress, and, in time, this will lead to intimal hyperplasia [24]. In a study by Nalesso et al. [25], the use of US to assess AVF needle positions showed that approximately 82% of the “traditional” needle placements were incorrect, mostly those closer to the arterio-venous anastomosis. By US-guided repositioning of the needle tip away from the vessel wall, endothelial stress may be reduced, thus limiting vessel damage. Moreover, by improving their cannulation technique, nurses approach more fistulas using the rope–ladder technique and abandon the ”area” technique, which may, in time, cause AVF aneurysms [26]. In the third item of the survey, “AVF POCUS had a positive effect on increasing my confidence in my vascular access cannulation skills”, there was a significant difference between the overall scores of the two surveys, with less favorable results upon follow-up. This might be due to the fact that in the cases where US-guided cannulations were performed, the procedure was led by the nephrologist who carried out the scan, indicated the puncture site, and supervised the process. Therefore, the US-guided technique, as implemented in our study, could have reduced the nurses’ decision-making process and, by consequence, their confidence. This result highlights the importance of POCUS training for nurses, not just physicians. In a study by Hill et al. [27] regarding cannulation confidence before and after POCUS training, the nursing staff showed a shift toward higher confidence levels in AVF management after being trained to use POCUS. The method employed in this study involved a theoretical program conducted by a Registered Vascular Technologist and an Accredited Medical Sonographer regarding the principles of US and VA assessment, followed by 6 h of face-to-face practical training. Additionally, the study included a patient questionnaire concerning their experience with POCUS. Most of the patients were very receptive to the method and were certain that it would improve their needling experience. Similarly, Chen et al. [28] conducted a prospective, 1-year study regarding nursing confidence levels pre- and post-US-guided cannulation training. They concluded that by implementing handheld US-guided cannulation, renal nurses had higher confidence levels and improved success rates. Currently, US-guided cannulation programs for nurses are not widely available, and most HD nurses are not familiar with the broad array of situations in which US may be helpful. Implementing educational programs regarding US-based techniques for nurses has proven its benefits for successful peripheral catheterization [29], performing transthoracic echocardiography [30], or in acute gerontological care [31].

The team at McGill [32] implemented a nurse-led VA surveillance clinic consisting of scheduled clinical and US monitoring of AVFs and AVGs. In their surveillance regime, not only did the nurse perform the US examinations, but they also triaged the referrals toward the treating vascular surgeon. The results of this program were assessed after a 3-year period, achieving a reduction in the number of thrombosed fistulas, fewer hospital days, and lower overall costs. Considering the existing data as well as the results of our study, the implementation of nurse-led POCUS AVF cannulation might be beneficial for increasing nurses’ confidence in VA puncturing. Duncanson et al. [33] performed a qualitative study regarding nephrology nurses’ perspectives when working with patients that experience needle distress. One of the viewpoints manifested in this article was that ongoing professional education is crucial and that proficiency in ultrasound-based cannulations may reduce unsuccessful cannulations.

The fourth item, “AVF POCUS is a tool for better vascular access teamwork”, was introduced to assess whether the nursing staff perceives this method as a contributor to forming a more cohesive vascular access team since all POCUS assessments were performed by the attending physician. Even though there was a slight decrease in the positivity of responses upon follow-up (11.4% disagreed), 88.6% of the nurses answered in agreement/full agreement. The importance of teamwork for vascular access care has not been extensively studied yet; however, a study performed by Gruss et al. [34] proved that introducing a multidisciplinary team monitoring program for AVFs led to lower rates of AVF thrombosis and fewer catheter insertions. In our survey, we observed that using AVF POCUS in the HD unit enhanced the work–team cohesiveness by increasing the support from physicians, which may lead to a better work environment and overall better patient-centered care.

Lastly, the fifth survey item, “AVF POCUS is an indispensable tool for AVF care”, was included to sum up the importance of implementing this method in the HD unit, and we recorded no differences in perception either at the initial survey or upon follow-up (all nurses were in agreement/full agreement). The current KDOQI (Kidney Disease Outcomes Quality Initiative) vascular access guidelines issued the statement that it “is reasonable to use ultrasound to help determine direction of flow and proper needle placement in the AV access of select patients as needed and performed by trained operators to prevent cannulation complications.” [1]. This statement implies that vascular ultrasound training should be performed, which is not a current standard practice yet in the HD units. This might explain why vascular access POCUS is not so widespread, even though the overall perception of the technique is positive.

The current study is the only long-term (5 years), prospective study regarding nurses’ perceptions of AVF POCUS, which offers a realistic perspective on the implementation of the technique in HD units. The main limitations of this study are that it is single-centered, so the viewpoints offered may not be generalizable, and the number of collected responses is low. Moreover, we acknowledge that some of the nurses who responded to the follow-up questionnaire differed partially from the initial ones, which may reduce the power of this prospective study. However, we emphasize that staff turnover is a reality in the dialysis unit setting and that the current study reflects the overall opinions of the nursing body.

## 5. Conclusions

The implementation of AVF POCUS in the HD unit was initially received positively, and the perception remained overall favorable at a 5-year follow-up. Our study highlights the importance of VA access to US training, not only from the perspective of better clinical outcomes but also from the subjective viewpoint of the nursing staff, who may benefit from an additional assessment tool. The overall perception was that AVF POCUS has helped with cannulation technique improvement and that it is an indispensable tool for VA assessment in the HD unit. Our study supports the active efforts to include AVF POCUS as standard practice in HD units, considering the satisfaction with which the technique was adopted by the nursing staff.

## Figures and Tables

**Table 1:** Demographic characteristics of both groups (age and years employed in dialysis expressed as mean +/- standard deviation, gender expressed in percentages).

	Initial Survey (*n* = 35)	Follow-Up Survey (*n* = 34)	*p*
Age (years) (mean, std)	36.7 ± 11.38	42.2 ± 12.45	<0.001
Years employed as dialysis nurse (years) (mean, std)	6.11 ± 3.78	8.51 ± 6.11	0.06
Gender (M/F) %	M = 11.4% F = 88.6%	M = 8.8%, F = 92%	0.7

**Table 2:** Comparative numbers (and percentages) of responses, upon initial survey and follow-up.

Question	5: Strongly Agree		4: Agree		3: Neutral		2: Disagree		1: Strongly Disagree	
	Initial	Follow-Up	Initial	Follow-Up	Initial	Follow-Up	Initial	Follow-Up	Initial	Follow-Up
AVF POCUS is helpful in the successful cannulation of the new/difficult AVFs	34 (97%)	30 (88%)	1 (3%)	4 (12%)	0 (0%)	0 (0%)	0 (0%)	0 (0%)	0 (0%)	0 (0%)
AVF POCUS helps to improve my cannulation technique	33 (94%)	28 (82%)	1 (3%)	4 (12%)	1 (3%)	2 (6%)	0 (0%)	0 (0%)	0 (0%)	0 (0%)
AVF POCUS had a positive effect in increasing my confidence in my vascular access cannulation skills	32 (91%)	18 (53%)	2 (6%)	14 (41%)	0 (0%)	2 (6%)	0 (0%)	0 (0%)	1 (3%)	0 (0%)
AVF POCUS is a tool for better vascular access teamwork	29 (83%)	22 (65%)	6 (17%)	8 (24%)	0 (0%)	0 (0%)	0 (0%)	4 (11%)	0 (0%)	0 (0%)
AVF POCUS is an indispensable tool for AVF care	27 (77%)	24 (71%)	7 (20%)	10 (29%)	1 (3%)	0 (0%)	0 (0%)	0 (0%)	0 (0%)	0 (0%)

**Table 3:** Comparative overall scores from the initial and follow-up surveys.

Question	Overall Initial Score	Overall Follow-Up Score	*p*
AVF POCUS is helpful in the successful cannulation of the new/difficult AVFs	4.97	4.77	0.15
2. AVF POCUS helped to improve my cannulation technique	4.88	4.68	0.26
3. AVF POCUS has a positive effect in increasing my confidence in my vascular access cannulation skills	4.82	4.38	0.0006
4. AVF POCUS is a tool for better vascular access teamwork	4.74	4.41	0.19
5. AVF POCUS is an indispensable tool for AVF care	4.82	4.72	0.23

## Data Availability

The raw data supporting the conclusions of this article will be made available by the authors on request.

## Public Involvement Statement

There was no public involvement in any aspect of this research.

## Guidelines and Standards Statement

This manuscript was drafted against the STROBE Statement (Vandenbroucke JP, von Elm E, Altman DG, Gotzsche PC, Mulrow CD, Pocock SJ, Poole C, Schlesselman JJ, Egger M. Strengthening the Reporting of Observational Studies in Epidemiology (STROBE): Explanation and Elaboration) for observational research.
